# Association of multi-metals with the risk of hypertension and the interaction with obesity: A cross-sectional study in China

**DOI:** 10.3389/fpubh.2023.1090935

**Published:** 2023-03-16

**Authors:** Shan Wu, Lvrong Li, Guiyuan Ji, Xiaohui Xing, Jiajie Li, Anping Ma, Yuan Wei, Dongwei Zhao, Huimin Huang, Wenjun Ma, Banghua Wu, Ming Dong, Tao Liu, Qingsong Chen

**Affiliations:** ^1^Guangdong Provincial Engineering Research Center of Public Health Detection and Assessment, School of Public Health, Guangdong Pharmaceutical University, Guangzhou, China; ^2^School of Public Health, Guangdong Pharmaceutical University, Guangzhou, China; ^3^Guangdong Provincial Institute of Public Health, Guangdong Provincial Center for Disease Control and Prevention, Guangzhou, China; ^4^Innovation Team of Environmental Health Assessment and Public Health Strategy, Guangzhou, China; ^5^Guangdong Province Hospital for Occupational Disease Prevention and Treatment, Guangzhou, China; ^6^Department of Public Health and Preventive Medicine, School of Medicine, Jinan University, Guangzhou, China; ^7^Disease Control and Prevention Institute of Jinan University, Jinan University, Guangzhou, China; ^8^National Medical Products Administration (NMPA) Key Laboratory for Technology Research and Evaluation of Pharmacovigilance, Guangdong Pharmaceutical University, Guangzhou, China

**Keywords:** multiple metals, hypertension, blood pressure, obesity, interaction, BKMR analysis

## Abstract

**Background:**

Environmental exposure to multiple metals have been inconsistently associated with hypertension. Obesity is an important independent risk factor for hypertension, and few studies have assessed the interaction between obesity and metals in this context. We aimed to clarify their association and interaction.

**Methods:**

This cross-sectional study included 3,063 adults from 11 districts or counties, Guangdong. We measured the whole blood levels of 13 metals and used multipollutant-based statistical methods to analyze the association of metals with hypertension. The interaction between metals and obesity on hypertension was assessed on additive and multiplicative scales.

**Results:**

Four metals (manganese, arsenic, cadmium, and lead) were significantly associated with hypertension risk, five metals (manganese, zinc, arsenic, cadmium, and lead) were related to elevated SBP levels, five metals (manganese, zinc, selenium, cadmium, and lead) were associated with elevated DBP levels in single-metal model. Manganese remained significantly related to hypertension risk [odds ratio, 1.35 (1.02–1.78)] after adjusting for these four metals. Significant positive dose-response relationships between manganese, arsenic, cadmium, lead and hypertension risk were observed (*P* for overall < 0.001, *P* for non-linearity > 0.05). Compared with those in the lowest quartile, participants in the highest manganese quartile had a 2.83 mmHg (95% Cl: 0.71–4.96) (*P*_FDR_ = 0.040) higher level of SBP. Individuals in the highest quartiles of zinc and lead had a 1.45 mmHg (0.10–2.81) (*P*_FDR_ = 0.033) and 2.06 mmHg (0.59–3.53) (*P*_FDR_ = 0.020) higher level of DBP, respectively. The negative interactions between cadmium, lead and obesity influences hypertension risk. BKMR analysis showed a significant joint effect of manganese, arsenic, cadmium and lead on hypertension when the concentrations of four metals were at or above their 55th percentile compared to their median values.

**Conclusions:**

The combined effect of four metals (manganese, arsenic, cadmium and lead) were associated with the prevalence of hypertension. Potential interaction effects of cadmium, lead and obesity on hypertension risk may exist. Further cohort studies in larger population are needed to clarify these findings.

## 1. Introduction

Hypertension is a major risk factors for cardiovascular disease and imposes a heavy burdens of societies internationally (1). It has been estimated that about 1.39 billion people worldwide have hypertension in 2010, and by 2030 the number of people with hypertension will reach to 1.56 billion (2, 3). Over the past decades, the prevalence of hypertension has been increasing in Chinese adults (4). It was reported that the prevalence of Chinese adult hypertension was 17.6% in 2002 (5), and it increased to 23.2% in 2015 (6). Hypertension has become a worldwide public health concern. However, the pathogenesis of hypertension has yet to be elucidated.

The traditional risk factors, including heredity, age, sex, BMI and unhealthy lifestyles, could only partially explain the causes of hypertension (7). Recently, accumulating epidemiological studies have evaluated the potential association between various metal levels from different biological samples and the prevalence of hypertension, but the results are inconsistent (8–10). A prospective study conducted in 3047 American Indian reported that the low-to-moderate levels of urinary cadmium (Cd) was positively correlated with hypertension (11). Nevertheless, a cross-sectional study from the Canadian Health Measures Survey (2007–2013) observed an inverse correlation of urine Cd with the risk of hypertension in current smokers, particularly female current smokers (12). The 2011–2016 US National Health and Nutrition Examination Survey (NHANES) confirmed a positive correlation between blood selenium (Se) levels and hypertension but not serum zinc (Zn) and copper (Cu) in American adults (13). A cross-sectional study conducted in the Cd-polluted and the unpolluted area of southwestern China unveiled that high blood iron (Fe) and lead (Pb) levels in polluted area and high blood manganese (Mg) and Fe in unpolluted area were related to increasing SBP and DBP levels (14).

In daily life, metals exist in almost all environmental media and people are often exposed to multiple metals simultaneously (15). Excessive visceral fat distribution, especially in obese individuals, can cause inflammatory reaction and endothelial damage, leading to hypertension (16). However, the effects of multiple-metal exposures on the risk of hypertension are largely unknown. A prospective cohort study in the Yangtze River region suggested that based on Bayesian kernel machine regression (BKMR), multiple metals [Cd, Cu, Mn, molybdenum (Mo), and Zn] had a significant joint effect on hypertension (10). Inter-metal interactions may alter the toxicity of single metals. Therefore, the co-exposure effect of multiple metals cannot be ignored in the study of metals.

As a widely known risk factor for higher blood pressure and hypertension, high BMI is reported to be associated with heavy metals (12, 17, 18). The 1999–2014 US NHANES reported that the negative interaction between Cd exposure and obesity influenced systolic hypertension risk (18), however, high urinary Cd levels were associated with significantly high blood pressure among overweight and obese Canadian women (12). Therefore, the effect of the interaction of heavy metals exposure and BMI on hypertension and blood pressure is worthy of further investigation.

In this context, we conducted a cross-sectional study from Guangdong Province located in southern China and measured the whole blood 13 metals levels as internal exposure. And the multipollutant-based statistical methods, including multivariate logistic regression, restricted cubic spline (RCS), the Bayesian kernel machine regression (BKMR) and interaction analysis, were applied to explore the joint effects of multiple metals on hypertension risk and their interaction with obesity.

## 2. Methods

### 2.1. Study population

This was a cross-sectional survey and the data obtained in the Guangdong Provincial Residents' Chronic Disease and Nutrition Surveillance Survey (2015). All participants were recruited from general communities and they were not exposed to metal pollutions or occupational factors. Briefly, 11 districts or counties were randomly selected, and about 300 participants were further selected in each district or county using a simple random sampling method. Finally, 3,029 participants aged 18 years or older were included between October 2015 and February 2016 after excluding 34 individuals who did not provide blood samples. The protocol of the present study was approved by the institutional review board of Chinese Center for Disease Control and Prevention (No. 201519-B), and all of subjects provided their written informed consent.

### 2.2. Data collection and outcome definition

A face-to-face questionnaire by a well-trained public health practitioner was applied to collect data. The details of the data collection methods have been described in previous study (19). Information on general demographic information, lifestyle, diseases, medication, and family history of hypertension were gathered. The anthropometric data, including systolic blood pressure (SBP), diastolic blood pressure (DBP), weight, height, and waist circumference (WC), were also measured using standard methods (19). Waist-to-height (WHt R) was calculated as waist (cm) divided by height (cm). The levels of fasting blood-glucose (FBG), total triglycerides (TG) and total cholesterol (TC) were also tested following a standard protocol (19).

Drinking status was classified based on alcohol consumption into current drinking (any alcohol consumption in the past 30 days) and not drinking. Smoking status was classified as “non-smokers,” “former smokers” (no tobacco use in the past 30 days) or “current smokers” (any tobacco use in the past 30 days).

Hypertension was defined as a self-reported physician diagnosis (confirmed with medical records from district-level or higher hospitals), or individuals with SBP ≥ 140 mmHg and/or DBP ≥ 90 mmHg for three times, or current use of antihypertensive medication (20). Body mass index (BMI) was classified into four categories following the standards of Chinese adults: low body weight, < 18.5 kg/m^2^; normal, ≥18.5 kg/m^2^, and < 24 kg/m^2^; overweight, ≥24 kg/m^2^ and < 28 kg/m^2^; obesity, ≥28.0 kg/m^2^. Individuals with central obesity were those with WC ≥85 cm for males or ≥80 cm for females (21).

### 2.3. Whole blood metal measurements

The whole blood concentrations of 13 metals (As; Cd; Cobalt, Co; Chromium, Cr; Cu; Mn; Mo; Nickel, Ni; Pb; Se; Thallium, Tl; Zn; Vanadium, V) were measured by inductively coupled plasma mass spectrometry (ICP-MS, Agilent 7500ce, Agilent Technologies, USA). The fasting blood samples were collected and stored at −80°C for subsequent detection following a standard detailed protocol (19). Before analysis, frozen whole blood samples were thawed at room temperature (22 ± 5°C). Two hundred and fifty microliter of the sample was diluted with 4.75 mL diluent [including 2.5 ml HNO_3_ (Guaranteed reagent, Fisher) and 0.5 ml TritonTMX-100 (Analytial reagent, sigma-alorich)], and the sample was further diluted to 1,000 ml with ultrapure water for final analysis. Trace Element Blood L-1~2 (Seronorm, Sero, Oslo, Norway) were applied as quality control samples, and quality control was run in each batch (25 samples). Besides, three blank samples (1% HNO_3_) were used to control the potential contaminations and set into each batch. Meanwhile, the spiked recovery method was used by measuring each sample twice and the recovery rates of all metals were from 90.0 to 110.0%. Once the measured values were suggested to be contaminated or differ from certified values, the instrument was recalibrated and the previous batch of samples was reanalyzed. The data were multiplied by the dilution factor to gain the final concentration. Measurements below the limit of detection (LOD) were replaced with LOD/$\sqrt{2}$. The LODs for the 12 metals ranged from 0.2 (Co, Cd, Tl) to 20.0 (Zn, Pb) μg/L. Since the proportion of participants whose whole blood Tl and Mo with concentrations lower than LOD was 88.7 and 48.04%, Tl and Mo was not further analyzed. For other metals, more than 70.91% of the samples had values above the LOD (Supplementary Table S1).

### 2.4. Statistical analysis

The demographic characteristics of all the subjects were described as percentages, means ± standard deviation (SD), or median with interquartile range (IQR). *T*-tests, chi-square test and the Wilcoxon rank sum were used based on data distribution. The levels of metals were transformed to natural logarithm (ln) to approximate a normal distribution. Pearson correlation test was used to evaluate the correlation between whole blood metals.

The blood metal level was divided into quartiles, and the lowest quartile (Q1) was assigned to be the referent group. The relationship between blood metal level and the risk of hypertension was evaluated by using multivariate logistic regression model with 11 districts or coastal/inland as fixed effect and the mixed effects logistic regression model with 11 districts or coastal/inland as a random intercept, respectively, and the optimal model was further selected for analysis. The results of model are shown in Supplementary Tables S2–S5. Multivariable linear model was applied to assessed the associations of metal levels in whole blood and BP/PP levels. Taking into account the potential deviation between taking antihypertensive drugs and blood pressure level, the BP level of participants using antihypertensive drugs increased by an additional constant of 10 mmHg (22). The covariates adjusted in the single- and multiple-metals models were age, sex, region and education level, drinking and smoking statue, family history of hypertension, antihypertensive use, BMI, TG, TC, LDL, HDL, HbAlc, UA (10, 23–26). The trend test was performed by taking the median of each metal quartile as a continuous variable in the model, and the false discovery rate (FDR) correction was applied to adjust trend *P*-values.

Restricted cubic spline (RCS) analyses were applied to further explore the dose-response relationship of whole blood metal level with hypertension risk, with the percentiles of five the ln-transformed metal concentrations to be the knots. Adjusted factors were same to multiple-metal models.

Bayesian Kernel Machine Regression (BKMR) based on Gaussian process regression was used to investigate the non-linear dose-response and interactions of metals on hypertension. Metals (Mn, As, Cd, and Pb) with significant or suggestive P trend (*P* trend < 0.05 or 0.05 < *P* trend < 0.10) in single-metal models were incorporated into BKMR model. Adjusted factors were same to multiple-metal models. First, four metals were classified into two groups based on the correlation coefficients. The conditional posterior inclusion probability (con PIP) was calculated to select the key metals for hypertension, and the threshold for PIP was 0.5 (27). Then, exposure-response function was performed to explore the association of individual metal with the risk of hypertension while holding the other four metals at median. Moreover, the joint effect of four metals on the risk of hypertension was estimated by comparing a particular percentile of multiple metals against their median value. Finally, an interaction of a particular metal and the remaining metals was estimated when the remaining metals were fixed at a particular percentile (25th, 50^th^, or 75th percentile). Sensitivity analyses for BKMR were performed by changing its smoothing parameters (b = 50 and b = 200).

Subgroup analysis and modeled interaction terms was used to examine the modification of associations in logistic regression model according to BMI (low body weight, normal, overweight, and obesity), WHt R (< 0.6 or ≥0.6), central obesity (yes or no), drinking status (drinking within 30 days, drinking before 30 days, never drinking) and smoke status (current smokers, former smokers, non-smokers). Subgroup analysis was limited to metals that predicted the outcome with *P* < 0.10 in the single-metal models.

All statistical analyses were performed with IBM SPSS 26.0 (Armonk, NY, USA) and R software (v.4.1.0). Two-tailed *P* < 0.05 was reckoned as statistically significant, while *P*-values in the range of 0.05–0.10 was suggestively significant.

## 3. Results

### 3.1. Characteristics of participants

After all individuals who did not provide blood samples (n=34) were excluded, 3,029 of the 3,063 eligible individuals were included in the final data analysis. As shown in Table 1, the percentages of non-hypertension and hypertension subjects were 64 and 36%, respectively. Compared to subjects without hypertension, those with hypertension were more likely to be female, older, former smoker and current alcohol consumers, live in island areas, had lower education level and higher SBP, DBP, WHt R, BMI, TC, TG, and FBG (all *P* < 0.05). The whole blood levels of Cu, Zn, Mo, Cd, Pb were significantly higher (all *P* < 0.05) in the hypertension group than in non-hypertension, while no significant differences were observed for other metals (all *P* > 0.05). Pearson correlation coefficients (*r*) of metals ranged from −0.412 to 0.834 (all *P* < 0.05, Supplementary Table S6).

**Table 1:** Base characteristics of the study population.

**Characteristics**	**Hypertension (*n* = 1,090)**	**Non-hypertension (*n* = 1,939)**	***P*-value**
Age (years)	62.0 (53.2, 69.3)	48.6 (36.3, 58.7)	< 0.001^*^
Sex, *n* (%)			0.021^*^
Male	524 (48.1)	848 (43.7)	
Female	566 (51.9)	1,091 (56.3)	
Region, *n* (%)			< 0.001^*^
Coastal	428 (39.3)	932 (48.1)	
Inland	662 (60.7)	1,007 (51.9)	
Education level, *n* (%)			< 0.001^*^
≤ 9 years	898 (82.4)	1,332 (68.7)	
9–12 years	136 (12.5)	371 (19.1)	
>12 years	56 (5.1)	236 (12.2)	
Family history of hypertension, *n* (%)			0.01^*^
Yes	293 (26.9)	440 (22.7)	
No	797 (73.1)	1,499 (77.3)	
Anti-hypertensive use, *n* (%)	301 (27.6)	0 (0)	NA
Central obesity, *n* (%)			< 0.001^*^
Yes	604 (55.4)	641 (33.1)	
No	486 (44.6)	1,298 (66.9)	
Smoking status^a^, *n* (%)			< 0.001^*^
Current smokers	289 (26.5)	505 (26.0)	
Former smokers	101 (9.3)	93 (4.8)	
Non-smokers	700 (64.2)	1,341 (69.2)	
Drinking status, *n* (%)			< 0.001^*^
Drinking within 30 days	250 (22.9)	498 (25.7)	
Drinking before 30 days	91 (8.3)	271 (14.0)	
Never drinking	749 (68.7)	1,170 (60.3)	
SBP (mmHg)	149.7 (141.7, 162.3)	120.3 (111.0, 128.0)	< 0.001^*^
DBP (mmHg)	85.7 (77.3, 92.3)	73.3 (67.7, 78.3)	< 0.001^*^
BMI (kg/m^2^)	24.1 (21.7, 26.5)	22.5 (20.5, 24.7)	< 0.001^*^
WHt R	0.53 (0.49, 0.57)	0.49 (0.45, 0.53)	< 0.001^*^
TC (mmol/L)	5.23 (4.63, 5.94)	4.93 (4.26, 5.54)	< 0.001^*^
TG (mmol/L)	1.21 (0.84, 1.86)	0.97 (0.69, 1.48)	< 0.001^*^
FBG (mmol/L)	5.38 (4.89, 6.00)	5.03 (4.67, 5.48)	< 0.001^*^
Blood metals (μg/L)			
V	0.95 (0.50, 1.54)	0.99 (0.54, 1.52)	0.253
Cr	4.90 (3.68, 6.49)	4.97 (3.79, 6.46)	0.408
Mn	13.83 (11.00, 17.96)	14.05 (10.69, 18.01)	0.886
Co	0.28 (0.18, 0.38)	0.28 (0.18, 0.40)	0.111
Ni	2.25 (1.21, 3.90)	2.23 (1.31, 4.00)	0.328
Cu	962.84 (860.73, 1070.81)	932.37 (839.59, 1044.96)	< 0.001^*^
Zn	5703.99 (4819.70, 6568.20)	5534.83 (4762.66, 6410.31)	0.006^*^
As	4.48 (2.71, 7.10)	4.43 (2.62, 7.05)	0.518
Se	165.36 (137.57, 197.17)	165.72 (139.19, 194.20)	0.797
Cd	2.54 (1.44, 4.58)	2.12 (1.25, 4.21)	< 0.001^*^
Pb	45.51 (30.41, 64.97)	37.08 (24.58, 55.90)	< 0.001^*^

BMI, body mass index; DBP, diastolic blood pressure; FBG, fasting plasma glucose; NA, non-available; SBP, systolic blood pressure; TC, total cholesterol; TG, triglycerides; WHt R, waist height ratio.

^*a*^Smoking status was classified as “non-smokers,” “former smokers” (Tobacco was used before but not now) or “current smokers” (Tobacco was used daily or occasionally).

^*^P < 0.05.

### 3.2. Whole blood metals and hypertension risk

In the single-metal model (Table 2), after adjusting for age, sex, region and education level, drinking and smoking status, family history of hypertension and antihypertensive use (Model 1), the multivariate-adjusted ORs (95% CIs) of hypertension were 1.40 (1.07, 1.82) for Mn and 1.44 (1.08–1.94) for As comparing the highest vs. the lowest quartiles of metals. After additionally adjusted for BMI, TC, TG and FBG (Model 2), the ORs (95% CIs) of hypertension were 1.42 (1.08–1.86) for Mn, 1.41 (1.04–1.91) for As, 1.42 (1.05–1.91) for Cd, and 1.38 (1.02–1.86) for Pb. Mn, As, and Cd evaluated in Model 2 had positive and significant increased trends of ORs for hypertension (all *P* trend < 0.05), Pb had suggestively significant increased trends (*P* trend < 0.10), but none of them was retained after FDR-adjustments (all *P*_FDR_ > 0.05).

**Table 2:** Adjusted odds ratios (95%CI) for hypertension according to quartile of whole blood metals exposure.

**Whole blood metals**	**Quartiles of whole blood metals (**μ**g/L)**				***P* trend ($P_{\text{FDR}}^{\text{d}}$)**
	**Q1**	**Q2**	**Q3**	**Q4**	
**Single-metal model**					
V	≤ 0.52	0.53–0.98	0.99–1.55	≥1.56	
*n* (case/total)	287/755	277/747	236/741	271/736	
Crude^a^	Ref	0.96 (0.78, 1.18)	0.76 (0.62, 0.94)^*^	0.95 (0.77, 1.17)	0.254 (0.339)
Model 1^b^	Ref	1.08 (0.84, 1.40)	0.93 (0.71, 1.22)	1.00 (0.77, 1.30)	0.716 (0.820)
Model 2^c^	Ref	1.10 (0.85, 1.43)	0.92 (0.70, 1.22)	1.00 (0.76, 1.31)	0.714 (0.857)
Cr	≤ 3.75	3.76–4.94	4.95–6.47	≥6.48	
*n* (case/total)	286/760	272/758	258/756	274/755	
Crude^a^	Ref	0.93 (0.75, 1.14)	0.86 (0.70, 1.06)	0.94 (0.77, 1.16)	0.104 (0.250)
Model 1^b^	Ref	1.06 (0.82, 1.37)	0.96 (0.74, 1.25)	1.04 (0.80, 1.34)	0.155 (0.660)
Model 2^c^	Ref	1.04 (0.80, 1.36)	0.92 (0.70, 1.21)	0.97 (0.74, 1.26)	0.614 (0.819)
Mn	≤ 10.79	10.80–13.96	13.97–17.99	≥18.00	
*n* (case/total)	263/758	296/757	261/757	270/757	
Crude^a^	Ref	1.22 (0.99, 1.51)	1.00 (0.81, 1.23)	1.05 (0.85, 1.30)	0.885 (0.947)
Model 1^b^	Ref	1.46 (1.13, 1.90)^*^	1.21 (0.93, 1.57)	1.40 (1.07, 1.82)^*^	0.286 (0.660)
Model 2^c^	Ref	1.48 (1.14, 1.93)^*^	1.23 (0.94, 1.61)	1.42 (1.08, 1.86)^*^	0.053^*^ (0.165)
Co	≤ 0.14	0.15–0.28	0.29–0.39	≥0.40	
*n* (case/total)	314/881	260/665	276/742	240/741	
Crude^a^	Ref	1.16 (0.94, 1.43)	1.07 (0.87, 1.31)	0.87 (0.70, 1.06)	0.009^*^ (0.036^*^)
Model 1^b^	Ref	1.21 (0.94, 1.55)	0.99 (0.77, 1.27)	1.08 (0.84, 1.40)	0.659 (0.820)
Model 2^c^	Ref	1.25 (0.97, 1.62)	1.01 (0.78, 1.31)	1.19 (0.92, 1.55)	0.411 (0.705)
Ni	≤ 1.26	1.27–2.24	2.25–3.96	≥3.97	
*n* (case/total)	283/758	262/764	282/753	263/754	
Crude^a^	Ref	0.88 (0.71, 1.08)	1.01 (0.82, 1.24)	0.90 (0.73, 1.11)	0.208 (0.312)
Model 1^b^	Ref	0.96 (0.74, 1.24)	1.01 (0.78, 1.31)	0.89 (0.69, 1.15)	0.330 (0.660)
Model 2^c^	Ref	0.97 (0.75, 1.26)	1.02 (0.79, 1.33)	0.89 (0.69, 1.16)	0.477 (0.716)
Cu	≤ 845.64	1845.65–945.24	945.25–1055.79	≥1055.80	
*n* (case/total)	238/758	248/757	296/757	308/757	
Crude^a^	Ref	1.07 (0.86, 1.32)	1.40 (1.14, 1.73)^*^	1.50 (1.21, 1.85)^*^	0.168 (0.312)
Model 1^b^	Ref	0.96 (0.74, 1.25)	1.13 (0.87, 1.46)	1.11 (0.85, 1.44)	0.482 (0.820)
Model 2^c^	Ref	0.93 (0.71, 1.22)	1.10 (0.84, 1.43)	1.13 (0.86, 1.48)	0.245 (0.588)
Zn	≤ 4778.14	4778.15–5578.83	5578.84–6481.80	≥6481.81	
*n* (case/total)	263/758	251/757	277/757	299/757	
Crude^a^	Ref	0.93 (0.76, 1.16)	1.09 (0.88, 1.34)	1.23 (1.00, 1.51)^*^	0.188 (0.312)
Model 1^b^	Ref	0.89 (0.68, 1.16)	1.02 (0.79, 1.33)	1.16 (0.90, 1.51)	0.569 (0.820)
Model 2^c^	Ref	0.83 (0.63, 1.08)	1.01 (0.77, 1.32)	1.07 (0.82, 1.39)	0.330 (0.660)
As	≤ 2.65	2.66–4.45	4.46–7.06	≥7.07	
*n* (case/total)	264/760	277/756	274/756	275/757	
Crude^a^	Ref	1.09 (0.88, 1.34)	1.07 (0.87, 1.32)	1.07 (0.87, 1.32)	0.373 (0.448)
Model 1^b^	Ref	1.16 (0.90, 1.50)	1.20 (0.92, 1.57)	1.44 (1.08, 1.94)^*^	0.020^*^ (0.240)
Model 2^c^	Ref	1.12 (0.86, 1.45)	1.18 (0.90, 1.55)	1.41 (1.04, 1.91)^*^	0.028^*^ (0.165)
Se	≤ 138.60	138.61–165.50	165.51–195.45	≥195.46	
*n* (case/total)	282/759	266/756	253/757	289/757	
Crude^a^	Ref	0.92 (0.75, 1.13)	0.85 (0.69, 1.05)	1.05 (0.85, 1.29)	0.947 (0.947)
Model 1^b^	Ref	1.03 (0.79, 1.33)	0.90 (0.69, 1.17)	1.23 (0.95, 1.59)	0.756 (0.820)
Model 2^c^	Ref	1.00 (0.76, 1.30)	0.86 (0.66, 1.13)	1.06 (0.81, 1.39)	0.933 (0.933)
Cd	≤ 1.30	1.31–2.28	2.29–4.37	≥4.38	
*n* (case/total)	240/760	252/755	303/758	295/756	
Crude^a^	Ref	1.09 (0.88, 1.35)	1.44 (1.17, 1.78)^*^	1.39 (1.12, 1.71)^*^	0.002^*^ (0.012^*^)
Model 1^b^	Ref	1.04 (0.80, 1.37)	1.08 (0.82, 1.42)	1.25 (0.93, 1.66)	0.273 (0.660)
Model 2^c^	Ref	1.14 (0.86, 1.51)	1.21 (0.91, 1.61)	1.42 (1.05, 1.91)^*^	0.023^*^ (0.165)
Pb	≤ 26.39	26.40–39.50	39.51–59.19	≥59.20	
*n* (case/total)	191/758	269/757	303/757	327/757	
Crude^a^	Ref	1.64 (1.31, 2.04)^*^	1.98 (1.59, 2.47)^*^	2.26 (1.82, 2.81)^*^	0.001^*^ (0.012^*^)
Model 1^b^	Ref	1.29 (0.97, 1.71)	1.27 (0.95, 1.69)	1.32 (0.98, 1.76)	0.279 (0.660)
Model 2^c^	Ref	1.26 (0.94, 1.68)	1.27 (0.95, 1.70)	1.38 (1.02, 1.86)^*^	0.055^†^ (0.165)
**Multiple-metal model** ^d^					
Mn	Ref	1.44 (1.11, 1.88)^*^	1.18 (0.90, 1.55)	1.35 (1.02, 1.78)^*^	0.129 (0.172)
As	Ref	1.11 (0.85, 1.44)	1.18 (0.90, 1.54)	1.34 (0.99, 1.81)	0.062^†^ (0.156)
Cd	Ref	1.12 (0.84, 1.49)	1.15 (0.86, 1.54)	1.33 (0.98, 1.81)	0.078^†^ (0.156)
Pb	Ref	1.21 (0.91, 1.63)	1.19 (0.88, 1.60)	1.24 (0.91, 1.68)	0.263 (0.263)

^*a*^Crude Model was the crude odds ratios (95% CI).

^*b*^Model 1 was adopted the general logistics regression with the region (coastal/inland) as fixed effect and adjusted for age, sex, region, education level, drinking status, smoking status, family history of hypertension and anti-hypertensive use.

^*c*^Model 2 was additionally adjusted for BMI, TC, TG and FBG except for covariates in Model 1.

^*d*^Metals that were significant in the single-metal model 2 were all included in multi-metal models, adjusted covariates were the same to single-metal model 2.

^*^P < 0.05, ^†^P < 0.10 and >0.05.

In the multiple-metal models that simultaneously included four metals, trends for As and Cd remained suggestively significant, but neither of them were retained after FDR-adjustments (both *P*_FDR_ > 0.10). And only the associations of whole blood Mn with hypertension risk remained significant, and the multivariate adjusted ORs (95% CIs) of the highest quartiles of metals was 1.35 (1.02–1.78) for Mn.

### 3.3. Whole blood metals and blood pressure

The associations of metals in whole blood with the levels of SBP and DBP were investigated in the multivariable linear regression model (Table 3). In the single-metal models, the positive trends of Mn, Cd, and Pb with SBP were found (all *P* trend < 0.05), and these trends were retained after FDR-adjustments (all *P*_FDR_ < 0.10). Compared with the reference group, participants in the highest Cr, Mn, As, Cd, and Pb groups had a 0.98 mmHg (95% CI, 0.49–1.47), 3.41 mmHg (95% CI, 1.32–5.50), 2.39 mmHg (95% CI, 0.09–4.69), 2.73 mmHg (95% CI, 0.40–5.05) and 2.77 mmHg (95% CI, 0.48–5.06) higher level of SBP, respectively. Increasing trends of Mn, Cd, Pb, and Zn quartiles with elevated DBP levels (both *P* trend < 0.05) were found, and the trends were remained after FDR-adjustments (all *P*_FDR_ < 0.05). Individuals in the highest group of Mn, Zn, Cd, and Pb had a 1.92 mmHg (95% CI, 0.61–3.23), 2.09 mmHg (95% CI, 0.77–3.41), 2.38 mmHg (95% CI, 0.92–3.83), and 2.63 mmHg (95% CI, 1.21–4.05) higher in DBP level, respectively. In the multi-metals models, significant increasing trends of Mn or Zn and Pb quartiles with SBP or DBP levels were observed, respectively, and the trends remained after FDR-adjustments (all *P*_FDR_ < 0.05). Participants in the highest quartiles of Mn or Zn and Pb had a 2.83 mmHg (95% CI, 0.71–4.96), 1.45 mmHg (95% CI, 0.10–2.81) and 2.06 mmHg (95% CI, 0.59–3.53) higher in SBP or DBP levels, respectively.

**Table 3:** Beta coefficients and 95% CI of blood pressures in generalized linear regression analysis.

**Whole blood metals**	**Quartiles of whole blood metals (**μ**g/L)**				***P* trend ($P_{\text{FDR}}^{\text{a}}$)**
	**Q1**	**Q2**	**Q3**	**Q4**	
**SBP**					
**Single-metal model** ^b^					
V	Ref	1.07 (−1.01, 3.15)	−0.65 (−2.84, 1.53)	0.15 (−1.99, 2.29)	0.768 (0.838)
Cr	Ref	−0.17 (−2.31, 1.96)	0.40 (−1.71, 2.51)	0.98 (0.49, 1.47)^*^	0.971 (0.971)
Mn	Ref	2.71 (0.66, 4.77)^*^	3.52 (1.46, 5.58)^*^	3.41 (1.32, 5.50)^*^	0.001^*^ (0.012^*^)
Co	Ref	2.28 (0.22, 4.33)^*^	0.28 (−1.72, 2.29)	1.11 (−0.93, 3.15)	0.546 (0.728)
Ni	Ref	0.69 (−1.37, 2.75)	1.05 (−1.02, 3.11)	−0.56 (−2.63, 1.50)	0.752 (0.838)
Cu	Ref	−0.55 (−2.62, 1.51)	1.65 (−0.44, 3.75)	0.93 (−1.20, 3.06)	0.233 (0.399)
Zn	Ref	−1.77 (−3.83, 0.29)	1.33 (−0.76, 3.41)	0.91 (−1.20, 3.03)	0.089^†^ (0.212)
As	Ref	0.87 (−1.18, 2.93)	0.42 (−1.67, 2.50)	2.39 (0.09, 4.69)^*^	0.060^†^ (0.180)
Se	Ref	0.38 (−1.68, 2.44)	0.69 (−1.37, 2.76)	1.67 (−0.42, 3.77)	0.106 (0.212)
Cd	Ref	1.71 (−0.36, 3.79)	2.80 (0.64, 4.95)^*^	2.73 (0.40, 5.05)^*^	0.013^*^ (0.064^†^)
Pb	Ref	1.49 (−0.64, 3.61)	2.28 (0.07, 4.50)^*^	2.77 (0.48, 5.06)^*^	0.016^*^ (0.064^†^)
**Multiple-metal model** ^c^					
Mn	Ref	2.51 (0.45, 4.56)^*^	3.11 (1.02, 5.19)^*^	2.83 (0.71, 4.96)^*^	0.008^*^ (0.040^*^)
Zn	Ref	−2.06 (−4.13, 0.01)	0.84 (−1.25, 2.94)	0.18 (−1.96, 2.31)	0.311 (0.311)
As	Ref	0.76 (−1.29, 2.81)	0.38 (−1.70, 2.47)	1.71 (−0.61, 4.02)	0.225 (0.281)
Cd	Ref	1.29 (−0.80, 3.38)	2.11 (−0.08, 4.31)	1.79 (−0.62, 4.19)	0.108 (0.180)
Pb	Ref	1.11 (−1.00, 3.22)	1.68 (−0.55, 3.92)	1.90 (−0.45, 4.24)	0.107 (0.180)
**DBP**					
**Single–metal model** ^b^					
V	Ref	−0.08 (−1.37, 1.21)	−0.67 (−2.02, 0.69)	−1.00 (−2.33, 0.33)	0.100 (0.200)
Cr	Ref	−0.53 (−1.85, 0.80)	−1.00 (−2.33, 0.33)	−0.08 (−1.40, 1.24)	0.783 (0.899)
Mn	Ref	1.37 (0.09, 2.65)^*^	1.92 (0.63, 3.21)^*^	1.92 (0.61, 3.23)^*^	0.003^*^ (0.009^*^)
Co	Ref	0.91 (−0.38, 2.20)	−0.21 (−1.47, 1.05)	0.33 (−0.94, 1.61)	0.973 (0.973)
Ni	Ref	−0.28 (−1.57, 1.00)	0.55 (−0.74, 1.85)	0.19 (−1.10, 1.48)	0.496 (0.744)
Cu	Ref	−0.17 (−1.45, 1.12)	0.45 (−0.86, 1.76)	−0.42 (−1.75, 0.91)	0.764 (0.899)
Zn	Ref	−0.14 (−1.43, 1.15)	0.95 (−0.35, 2.24)	2.09 (0.77, 3.41)^*^	0.001^*^ (0.004^*^)
As	Ref	0.17 (−1.13, 1.46)	0.06 (−1.25, 1.36)	1.25 (−0.18, 2.69)	0.144 (0.247)
Se	Ref	−0.20 (−1.48, 1.08)	0.14 (−1.15, 1.42)	1.17 (−0.14, 2.48)	0.076^†^ (0.182)
Cd	Ref	0.98 (−0.31, 2.28)	1.41 (0.06, 2.75)^*^	2.38 (0.92, 3.83)^*^	0.001^*^ (0.004^*^)
Pb	Ref	1.25 (−0.07, 2.56)	2.33 (0.96, 3.70)^*^	2.63 (1.21, 4.05)^*^	< 0.001^*^ (0.001^*^)
**Multiple-metal model** ^c^					
Mn	Ref	1.02 (−0.25, 2.30)	1.46 (0.17, 2.76)^*^	1.10 (−0.23, 2.42)	0.081^†^ (0.101)
Zn	Ref	−0.40 (−1.70, 0.89)	0.62 (−0.70, 1.93)	1.45 (0.10, 2.81)^*^	0.013^*^ (0.033^*^)
Se	Ref	−0.26 (−1.54, 1.02)	−0.40 (−1.70, 0.90)	0.09 (−1.27, 1.44)	0.979 (0.979)
Cd	Ref	0.57 (−0.73, 1.87)	0.63 (−0.74, 1.99)	1.42 (−0.07, 2.91)	0.079^†^ (0.101)
Pb	Ref	1.02 (−0.30, 2.33)	1.85 (0.46, 3.25)^*^	2.06 (0.59, 3.53)^*^	0.004^*^ (0.020^*^)

^*a*^False Discovery Rate (FDR) adjusted p-values.

^*b*^Model was adjusted for age, sex, region, education level, drinking status, smoking status, family history of hypertension, BMI, TC, TG, and FBG.

^*c*^Five whole blood metals were simultaneously included in generalized linear regression with adjustment for the covariates used in the single-metal models.

^*^P < 0.05, ^†^P < 0.10 and >0.05.

### 3.4. Dose-response relationships of metal level and hypertension risk

**Figure 1:**
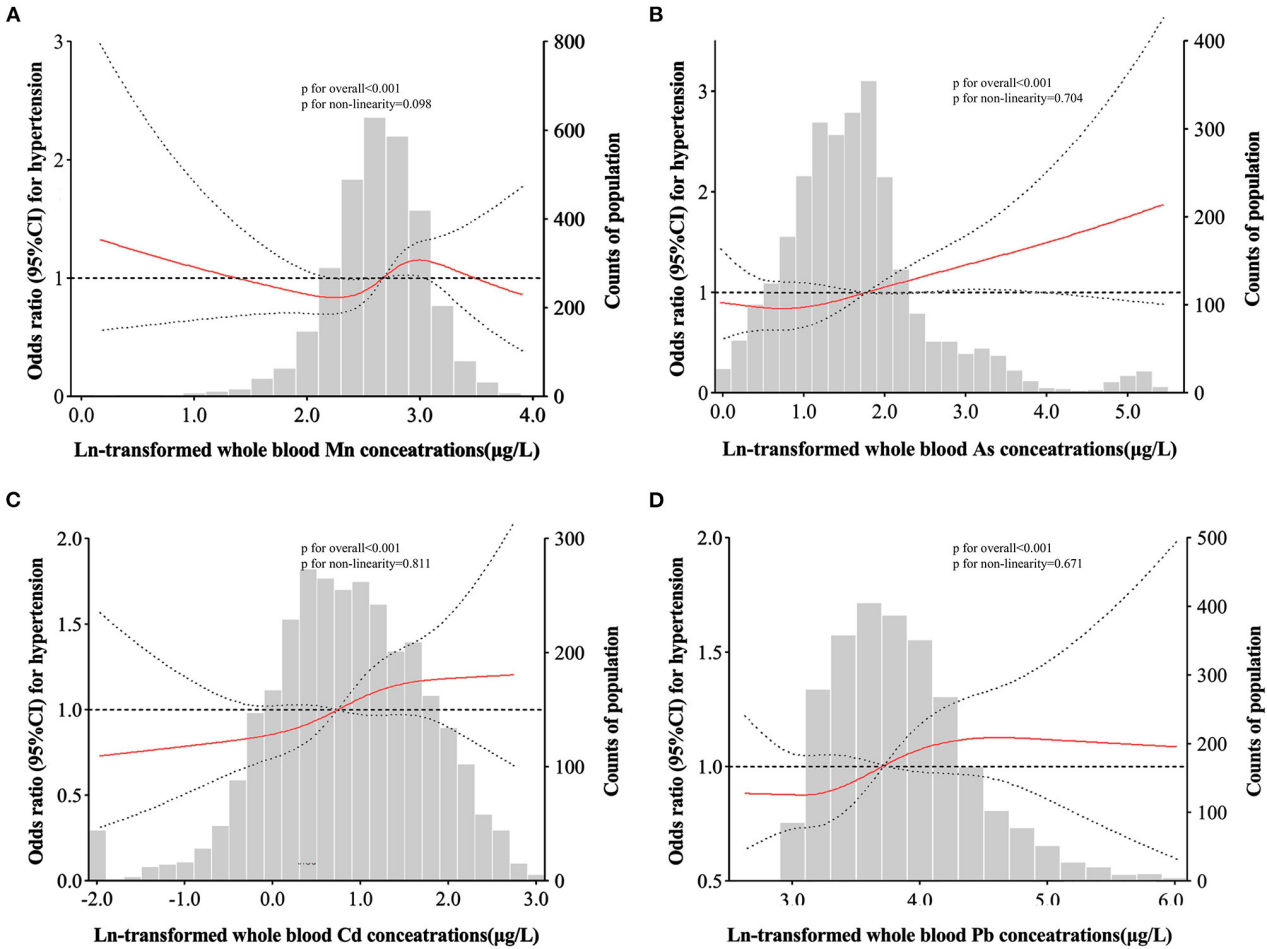
Adjusted restricted cubic spline (RCS) for the association between blood metals and hypertension. The red lines indicate adjusted odds ratios and the black lines represent adjusted odds ratios [95% CI] based on RCS for the log-transformed levels of Mn **(A)**, As **(B)**, Cd (**C)**, and Pb **(D)** in the single-metal models, with the reference value was set at the 10th percentile. Adjustment factors were age, sex, region, education level, drinking status, smoking status, family history of hypertension, antihypertensive use, BMI, TC, TG, and FBG.

For whole blood Mn levels, RCS analyses showed a wave shaped associations with the risk of hypertension (*P* for overall < 0.001, *P* for non-linearity = 0.098). The positive linear dose-response relationships between whole blood As, Cd, Pb and the hypertension risk were presented (all *P* for overall < 0.001, *P* for non-linearity of 0.704, 0.811, and 0.671, respectively) (Figure 1).

### 3.5. BKMR analyses

**Figure 2:**
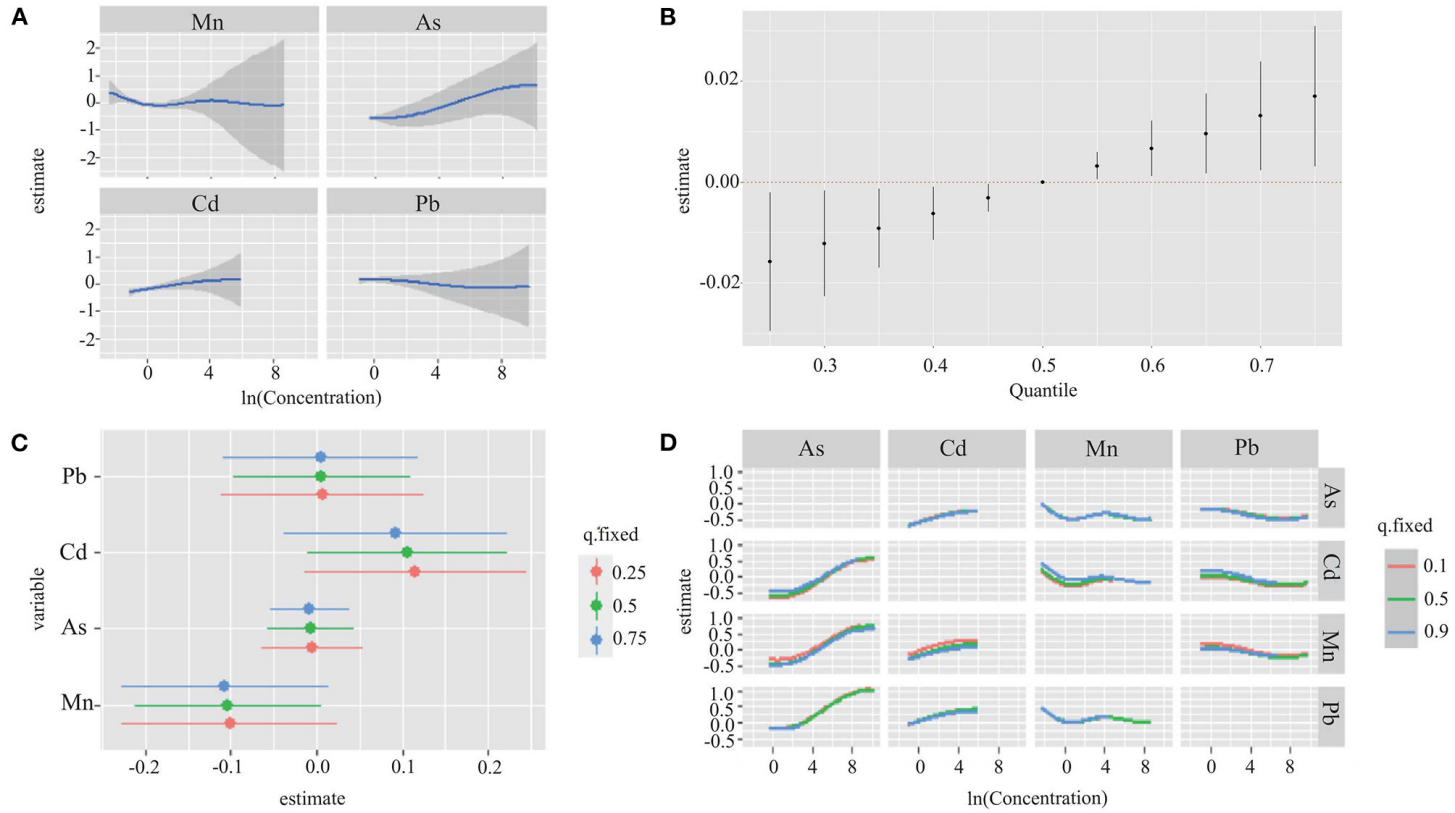
Joint effect of the four metals (Mn, As, Cd, and Pb) on hypertension risk by using BKMR model. The results were adjusted for age, sex, region, education level, drinking status, smoking status, family history of hypertension, antihypertensive use, BMI, TC, TG, and FBG. **(A)** Univariate exposure-response functions and 95% CI for association between single metal exposure when other metals exposure are fixed at the median. **(B)** Overall effect of the mixture estimates and 95% CI. **(C)** Single metal association (estimate and 95% CIs). This plot compares hypertension risk when a single metal was at the 75th vs. 25th percentile, when all the other metals were fixed at either the 25th, 50th, or 75th percentile. **(D)** Bivariate exposure response functions for each of the metal presented on the right longitudinal axis when the other metal presented on the upper coordinate axis holding at different quantiles (25th, 50th, and 75th) and other two metals were held at the median.

Figure 2A found a linear relationship between exposure to single metal (Mn, As, Cd, and Pb) and hypertension risk when other three metals exposure were fixed at the median. The conPIP of As, Cd, Mn and Pb was 0.67, 0.44, 0.40, and 0.44, respectively. A significant joint effect of four metals on the risk of hypertension was found when all metals were at or above their 55th percentile compared with their median values (Figure 2B). However, no significant association between single metal (Mn, As, Cd, and Pb) and hypertension risk (50th vs. 25th) was found when other three metals were fixed at different percentiles (25th, 50th, or 75th) (Figure 2C). No interaction effect was found among the four metals in the bivariate exposure-response analysis (Figure 2D). In addition, sensitivity analyses found that our findings were not sensitive to the choice of smoothing parameter.

### 3.6. Subgroup and interaction analyses

In normal BMI subgroups, significantly increased trends of OR for hypertension with Mn, Cd exposure were observed (both *P* trend < 0.05). Similar trends of OR for hypertension with As, Pb exposure were presented in overweight subgroup (Supplementary Table S7). Nevertheless, no trends of OR for hypertension with four metals (Mn, Cd, As, and Pb) exposure in subgroups of WHt R, central obesity, smoking and drinking status were observed (data not shown). To further explore the effect of the potential interaction between blood metals and BMI on hypertension, whole blood metals and BMI were categorized into two categories [blood metals: ≥geometric mean (GM) vs. < GM; and BMI: ≥28 kg/m^2^ indicating obesity vs. < 28 kg/m^2^ indicating non-obesity]. On the additive scale, remarkable negative interaction between BMI and Cd, Pb on hypertension were discovered [RERI = −2.35 (95% CI, −4.58, −0.12); RERI = −1.17 (95% CI, −2.28, −0.06), respectively] (Table 4). Similar findings were obtained on the multiplicative scale [OR = 0.50 (95% CI, 0.26–0.95); OR = 0.53 (95% CI, 0.28–1.00), respectively].

**Table 4:** Effect of interaction between whole blood metal and BMI on hypertension risk.

**Whole blood metals (μg/L)**	**Odds ratios (95% CI)**		
	**BMI**		**Within strata of metals**
	<**28**	≥**28**	
**Mn**			
Lower 50%	1.00	1.10 (0.90, 1.34)	2.17 (1.56, 3.89)^*^
Upper 50%	2.49 (1.57, 3.93)^*^	1.83 (1.16, 2.90)^*^	1.67 (1.04, 2.69)^*^
Within strata of BMI	1.10 (0.90, 1.34)	0.83 (0.44, 1.56)	
**As**			
Lower 50%	1.00	1.14 (0.92, 1.41)	1.67 (1.01, 2.74)^*^
Upper 50%	1.69 (1.04, 2.74)^*^	2.71 (1.73, 4.24)^*^	2.44 (1.57, 3.80)^*^
Within strata of BMI	1.14 (0.92, 1.42)	1.78 (0.87, 3.67)	
**Cd**			
Lower 50%	1.00	1.21 (0.97, 1.50)	2.99 (1.87, 4.78)^*^
Upper 50%	2.81 (1.79, 4.43)^*^	1.75 (1.09, 2.82)^*^	1.46 (0.91, 2.33)
Within strata of BMI	1.21 (0.97, 1.51)	0.49 (0.23, 1.02)	
**Pb**			
Lower 50%	1.00	1.18 (0.95, 1.46)	3.04 (1.91, 4.84)^*^
Upper 50%	2.73 (1.74, 4.29)^*^	1.77 (1.10, 2.84)^*^	1.35 (0.85, 2.14)
Within strata of BMI	1.17 (0.94, 1.45)	0.78 (0.39, 1.56)	

Measure of interaction on additive scale: Cd, RERI (95% CI) = −2.35 (−4.58, −0.12); Pb, RERI (95% CI) = −1.17 (−2.28, −0.06). Measure of interaction on multiplicative scale: Cd, ratio of ORs (95% CI) = 0.50 (0.26, 0.95); Pb, ratio of ORs (95% CI) = 0.53 (0.28, 1.00). Model was adjusted for age, sex, district, education level, drinking status, smoking status, family history of hypertension, anti-hypertensive use, TC, TG, FBG, and four metals in the multi-metal model.

^*^P < 0.05.

## 4. Discussion

In this study, we evaluated the association of 13 blood metals with risk of hypertension and blood pressure in the south Chinese general population by various statistical models. The findings showed that positive trends for increased odds of hypertension with increasing Mn, As, Cd, and Pb quartiles, and these trends were further confirmed in RCS analysis. Increasing Mn, Cd, and Pb quartiles were associated with elevated SBP levels, and increasing Mn, Zn, Cd, and Pb quartiles were associated with elevated DBP levels. BKMR analyses indicated a positive joint effect of mixture of four metals (Mn, As, Cd, and Pb) on hypertension risk.

### 4.1. Manganese (Mn)

Manganese (Mn) is an essential dietary element and a critical component in dozens of proteins and enzymes that involves several biological processes, including carbohydrate and lipid metabolism, growth and reproduction, and formation of tissues (28). Overexposure to Mn can induce severe neurological damage manganese toxicity (29). Despite it is considered as a toxic heavy metal, Mn could play a potential role in controlling blood pressure due to its anti-oxidative function (30). However, the accumulation of Mn may cause inflammation and endothelial dysfunction, leading to elevated blood pressure or the formation of high blood pressure (31). Besides, Mn exposure can inhibit myocardial contraction, dilates blood vessels and induces hypertension (32). The relationship between the body burden of Mn and blood pressure or hypertension remains somewhat controversial. A cross-sectional study of 957 participants from the Gulf Long-Term Follow-up Study showed that Mn was positively associated with the risk of hypertension (9), while a negatively association of urinary Mn with blood pressure was found in another study (8). Another cross-sectional study included 2,597 Chinese adults with hypertension showed that blood Mn levels were associated with age, region and season, but not with SBP (β = −0.01, *P* > 0.05) and DBP (β = 0.00, *P* > 0.05) (33). In the present study, no significant differences in whole blood Mn levels between hypertensive and non-hypertensive subjects were found, however, a positive trend and association of blood Mn with the risk of hypertensive, levels of SBP and DBP were observed in single-metal model (both *P* trend < 0.05), and this trend is maintained in levels of SBP and DBP after FDR-adjustments (both *P*_FDR_ < 0.10). Similar results were observed in a recent study (34). In the multiple-metal model, the association of blood Mn with hypertensive risk and SBP level were remained. In subgroup analysis, the risk of hypertension in High Mn + BMI ≥ 28 kg/m^2^ group was 1.83-folds comparing with the Low Mn + BMI < 28 kg/m^2^ group, but no obvious interaction on hypertension was observed. Taken together, the association between Mn and blood pressure or hypertension remain inconclusive. Further investigations are needed to validate our findings, and establish a standard biomarker of exposure to Mn.

### 4.2. Cadmium (Cd)

Cadmium (Cd) is a hazardous heavy metal with a long biological half-life of 10–30 years that may exert toxic effects on the human kidneys, liver, bones and respiratory system (11). The concentration of Cd in blood is regarded as internal exposure biomarkers, which can reflect cumulative exposure. And blood Cd with a half-life of 3–4 months is more appropriate to reflect short-term fluctuations in exposure (35, 36). Although the studies on the association of Cd exposure with hypertension are increasing, inconsistencies among studies still remain. The most common mechanisms associated with Cd exposure and the development of hypertension are similar to As (37). Cd may have direct vascular effects, it can inhibit endothelial nitric oxide synthase in blood vessels, thereby reducing endothelial relaxation by blocking cholinergic action and ultimately inducing hypertension (38). Cd may also lead to elevated vasoconstriction by increasing half-life of noradrenaline in vascular smooth muscle tissue (39, 40). Acute Cd exposure may activate the renin–angiotensin system (RAS), increase local release of angiotensin II and elevate activity of COX-2 and NADPH oxidase, and contribute to increased peripheral blood resistance with consequent genesis and maintenance of hypertension (39). Xu et al. (26) found no association between airborne Cd exposure and hypertension risk in the Sister Study. However, a systematic review reported that a positive association between blood Cd levels and blood pressure and/or hypertension was identified, while the association of urinary Cd remained uncertain (41). In the present study, Cd in whole blood was also found to be related to increased odds of hypertension and elevated SBP and DBP level in the single-metal model, but these relationships disappeared in the multiple-metal model, which could be due to the significant correlation between whole blood Cd and Zn (*r* = 0.146). We also found a negative interaction between whole blood Cd and subjects with BMI ≥ 28 kg/m^2^. High BMI has been reported to be associated with low level of Cd and other heavy metals (12, 18), but the relationships between Cd exposure and BMI are not consistent, which may be attributed to differences in exposure levels and the specific marker (42). Alexandre et al. (43) reported that no correlation of urine Cd level with blood pressure and hypertension in the general population of France, but a negative correlation of urine Cd with hypertension in obese subjects, chronic renal function and current smokers in the stratified model. The 1999–2014 NHANES suggested that the negative interaction between Cd exposure and obesity influenced systolic hypertension risk (18). Therefore, the potential mechanisms underlying the interaction of obesity/BMI and Cd exposure on hypertension or blood pressure are needed to further investigation.

### 4.3. Lead (Pb)

Lead (Pb) is an important heavy metal that is widely used in several applications, especially in industry, but it has no biological role in humans. Exposure to Pb produces various deleterious effects on the hemopoietic, renal, reproductive and central nervous system (44). Oxidative stress is recognized as one of the key mechanism of Pb-induced toxicity. Exposure to Pb promotes oxidative stress by enhancing the production of reactive oxygen species (ROS) or reducing the activity of antioxidant enzymes (45). Beside, Pb can reduce the availability of NO, which is an endogenous catalyst of several biochemical processes and plays an key role in the regulation of cardiovascular system, and lead to endothelial dysfunction (46). Epidemiological studies have shown that Pb exposure can increase the risk of hypertension (47–49). The 1999–2016 US NHANES demonstrated that blood lead level was not associated with hypertension [OR, 1.002 (0.983–1.021)], but positive associations of the blood Pb levels with SBP and DBP levels were found in non-Hispanic whites and non-Hispanic blacks who did not take antihypertensive, respectively (50). A cross-sectional study included 948 Brazilian adults with aged ≥40 showed that a positive trend and association of the blood lead level with the risk of hypertension or levels of SBP and DBP after adjusting the covariates (51). In this study, an increasing trend and association of whole blood Pb with the risk of hypertension and levels of SBP and DBP were found in single-metal model, and this trend and association of DBP level were maintained in multiple-metal model, similar to previous studies (52, 53). In subgroup analysis, compared with the Low Pb + BMI < 28 kg/m^2^ subgroup, the risk of hypertension in High Pb + BMI < 28 kg/m^2^ subgroup and High Pb + BMI ≥ 28 kg/m^2^ subgroup increased by 173 and 77%, respectively. Significantly negative interaction between blood Pb and BMI (only that of obesity but not overweight) was found. Conversely, Swayze et al. (54) found an interaction between high Pb levels and non-obese subjects (BMI < 30 kg/m^2^). This may be due to inconsistent results due to different definitions of obesity. There are several possible mechanisms by which lead exposure and obesity negatively interact with hypertension. One is that acute or chronic lead exposure does not destroy TC, but may reduce TC levels, which may be the reason why non obese individuals with high lead level have a higher risk of hypertension than obese individuals (55, 56). The Other is that Pb exposure may damage the gastrointestinal function and lead to weight loss (57). In addition, Pb exposure can reduce the basal level of cortisol and possibly reduce the risk of obesity by destroying the hypothalamic-pituitary-adrenal axis (58), which is consistent with the negative interaction between lead and obesity found in this study. However, the mechanism of interaction between lead and obesity on hypertension still needs more sufficient evidence to support.

### 4.4. Other metals

Arsenic (As) is a non-essential trace metal that has been classified as a human carcinogen (59). Long-term exposure to As by ingesting water, food and air pollutants may be associated with diabetes, diabetes, reproductive disorders, skin diseases, kidney diseases, cardiovascular diseases and cancer (60). A meta-analysis included eight studies indicated a positive association of As exposure with the risk of hypertension (61). After adjusted for age, sex and smoking status, a significant relationship between As level in the hair and the increased risk of hypertension was observed [OR, 2.0 (1.2–3.3); *P* < 0.05] (62). A cohort study conducted in Spanish male adolescents suggested that urinary As was associated with a slight elevation in SBP [0.70 mmHg (0.11–1.29)], per each 50% increase in metal concentrations) and an increased risk of elevated SBP (≥120 mmHg) [OR, 1.28 (1.04–1.56); *P* < 0.05] (63). Similarly, our findings showed that significant associations of blood As level with hypertension risk or SBP level in single-metal model after adjusting blood pressure-related covariates. In contrast, a null effect of As exposure in urinary on hypertension in a Chinese general population from a cross-sectional study was found (34). Blood As should be used as an internal exposure biomarker due to the metabolites of inorganic As could directly interact with target organs (64). The most common mechanisms underlying hypertension associated with As include oxidative stress, impaired nitric oxide (NO) signaling, inflammation, renal damage, altered vascular response to neurotransmitters and disturbed vascular muscle Ca^2+^ signaling, and interference with the renin-angiotensin system (37, 65, 66). Beside, As is a potential obesogen that adversely affect the basic metabolic functions of adipocytes by diminishing pre-adipocytes adipogenesis and increasing size of mature adipocytes (67). In our subgroup analysis, the risk of hypertension in High As + BMI ≥ 28 kg/m^2^ subgroup was 2.71-folds higher comparing with the Low As + BMI < 28 kg/m^2^ subgroup, additionally, whole blood As level and obesity had a synergistic effect on the risk of hypertension. A previous study has reported that measurements of body size, especially BMI, are associated with As metabolism biomarkers and this association may be related to obesity, fat free mass or body size (68). Accumulating evidence has presented that the risks of As-induced disease are significantly higher in obese individuals (69). Future epidemiological studies of As should consider BMI as a potential modifier for As-related diseases.

Zinc (Zn) is a basic chemical element for humans that widely involved in physiological processes, including protein, lipid, nucleic acid metabolism and gene transcription (70). Zn deficiency has been studied a lot, however, excess zinc may also exert toxic effects (71). Excessive Zn intake can cause oxidative stress and increase of ROS such as superoxide and peroxynitrite in the blood vessel wall, weaken the effect of vasodilator NO, which may play a role in inhibiting the increase of blood pressure by activating guanylate cyclase/cyclic guanosine monophosphate (CGMP) pathway, and ultimately lead to the increase of blood pressure (72). Currently, limited reports are available on the association between Zn and blood pressure, and the conclusions are still controversial. It may be due to differences in study subjects and internal exposure markers. A cross-sectional study included 40 obese Korean women aged 19–28 years (BMI ≥ 25 kg/m^2^) revealed that dietary Zn level was negatively correlated with SBP level, but serum and urine Zn levels were not significantly related to blood pressure (73). A prospective cohort study of 1,303 adults from the Yangtze River region of China reported a significant increased trend of Zn with the risk of hypertension in multiple-metals models (10). A cross-sectional study conducted in 823 adults from the physical examination center of the Union Hospital in Wuhan found that the risk of hypertension in the highest urine Zn quartiles had a 4.2-fold (95% CI:1.7–10.0) higher compared with the lowest quartiles (34). In this study, whole blood Zn concentration of participants with hypertension was significantly higher than that of non-hypertension (5703.99 vs. 5534.83 μg/L). A positive trend and association of blood Zn with DBP levels in single- and multiple-metals models was observed, and this trend remained after FDR adjustments. In the future, large-scale clinical trials and longitudinal studies are demanded to investigate the possibility of causality between Zn exposure and blood pressure.

### 4.5. Advantages and limitations

This study has several strengths that could improve the robustness of our findings. First, this is a relatively large Chronic Disease and Nutrition Surveillance Survey dataset (3,029 subjects), which ensures the adequate statistical power to test the associations between multiple metals and the risk of hypertension. Secondly, the multipollutant-based statistical methods, including multivariate logistic regression, RCS, BKMR and interaction analysis, were applied to explore the assocation of multiple metals with the risk of hypertension and their interaction with obesity, which could decrease the risk of misclassification bias. Third, the large sample size allowed us to adjust for many covariates including demographic characteristics, lifestyle behaviors, anthropometric features, socioeconomic status and other chronic diseases, which reduced the risk of confounding bias.

Several limitations should also be considered when interpreting the result. First, measurements of 13 metals in the same whole blood sample might increase measurement errors and the false positive rates, although FDR-method had been applied in our study. Secondly, we did not collect the information of dietary habit such as salt and sugar intake, while diet is an important factor that influences both levels of blood pressure and metals. However, the sensitivity analysis further confirmed the robustness of our findings. Third, this study was a cross-sectional study, the causal association between multiple metals and the risk of hypertension needs to be confirmed in further prospective studies.

## 5. Conclusions

In summary, this study indicated that higher levels of whole blood Mn, As, Cd, and Pb might be associated with the risk of hypertension, higher whole blood levels of Mn, Zn, As, Cd, and Pb might be related to elevated SBP levels, and higher whole blood levels of Mn, Zn, Se, Cd, and Pb might be associated with elevated DBP levels in the general population of South China. A significant joint effect of four metals (Mn, As, Cd, and Pb) were associated with the risk of hypertension. Moreover, the negative interactions between blood Cd, Pb levels and obesity influences the risk of hypertension. These findings extend our understanding of the relationship between exposure to multiple heavy metals and hypertension. The preventive strategies on heavy metal exposure may also contribute to the prevention of hypertension.

## Data availability statement

The raw data supporting the conclusions of this article will be made available by the authors, without undue reservation.

## Ethics statement

The protocol of the present study was approved by the Institutional Review Board of Chinese Center for Disease Control and Prevention (No. 201519-B), and all of subjects provided their written informed consent.

## Author contributions

SW, LL, and GJ made a substantial contributions to conceptualization, methodology, formal analysis, and writing—original draft. XX was involved in data curation. JL, AM, YW, DZ, and HH completed the methodology and data collection. WM contributed to the review, editing, and supervision. BW made contributions to project and resource management. MD, TL, and QC have made considerable contributions to funding acquisition, supervision, writing review, editing, and project administration. All authors contributed to the article and approved the submitted version.
