# Supplementary material for: Association of multi-metals with the risk of hypertension and the interaction with obesity: A cross-sectional study in China
Source: Front Public Health. 2023 Mar 16;11:1090935. doi: 10.3389/fpubh.2023.1090935 (PMC10063192; doi:10.3389/fpubh.2023.1090935)
Supplement: Supplementary file 1 [file Data_Sheet_1.pdf]

**Supplementary Table 1 Limits of detection, percentages of samples below detection limits (n=3029).**

| Plasma metals(μg/L) | LOD | Total No. (%) <LOD [a] |
|---|---|---|
| Vanadium (V) | 0.5 | 710 (23.44) |
| Chrome (Cr) | 1 | 49 (1.62) |
| Manganese (Mn) | 0.5 | 10 (0.33) |
| Cobalt (Co) | 0.2 | 881 (29.09) |
| Nickel (Ni) | 0.8 | 506 (16.71) |
| Copper (Cu) | 10 | 0 (0.00) |
| Zinc (Zn) | 20 | 0 (0.00) |
| Arsenic (As) | 1 | 183 (6.04) |
| Selenium (Se) | 2 | 63 (2.08) |
| Molybdenum (Mo) | 1 | 1455 (48.04) |
| Cadmium (Cd) | 0.2 | 44 (1.45) |
| Thallium (Tl) | 0.2 | 2692 (88.87) |
| Lead (Pb) | 20 | 427 (14.1) |

Note: LOD, limit of detection. No., number.

**Supplementary Table 2 Adjusted odds ratios (95%CI) for hypertension according to quartile of whole blood metals exposure.**

| Whole blood metal | Quartiles of whole blood metals (μg/L) | | | |
|---|---|---|---|---|
| | Q1 | Q2 | Q3 | Q4 |
| V | ≤ 0.52 | 0.53 ~ 0.98 | 0.99 ~ 1.55 | ≥ 1.56 |
| n (case/total) | 287/755 | 277/747 | 236/741 | 271/736 |
| Model 1[a] | Ref | 1.09 (0.83, 1.42) | 0.86 (0.65, 1.15) | 0.89 (0.68, 1.18) |
| Model 2[b] | Ref | 1.07 (0.82, 1.39) | 1.07 (0.82, 1.39) | 0.90 (0.68, 1.17) |
| Cr | ≤ 3.75 | 3.76 ~ 4.94 | 4.95 ~ 6.47 | ≥ 6.48 |
| n (case/total) | 286/760 | 272/758 | 258/756 | 274/755 |
| Model 1[a] | Ref | 0.98 (0.75, 1.29) | 0.84 (0.64, 1.11) | 0.88 (0.67, 1.16) |
| Model 2[b] | Ref | 0.96 (0.74, 1.25) | 0.96 (0.74, 1.25) | 0.88 (0.67, 1.15) |
| Mn | ≤ 10.79 | 10.80 ~ 13.96 | 13.97 ~ 17.99 | ≥ 18.00 |
| n (case/total) | 263/758 | 296/757 | 261/757 | 270/757 |
| Model 1[a] | Ref | **1.45 (1.11, 1.90)** | 1.19 (0.91, 1.57) | 1.31 (0.99, 1.73) |
| Model 2[b] | Ref | **1.44 (1.11, 1.88)** | 1.44 (1.11, 1.88) | 1.30 (0.99, 1.71) |
| Co | ≤ 0.14 | 0.15 ~ 0.28 | 0.29 ~ 0.39 | ≥ 0.40 |
| n (case/total) | 314/881 | 260/665 | 276/742 | 240/741 |
| Model 1[a] | Ref | 1.26 (0.97, 1.64) | 1.01 (0.77, 1.31) | 1.14 (0.88, 1.49) |
| Model 2[b] | Ref | 1.26 (0.98, 1.64) | 1.26 (0.98, 1.64) | 1.14 (0.88, 1.49) |
| Ni | ≤ 1.26 | 1.27 ~ 2.24 | 2.25 ~ 3.96 | ≥ 3.97 |
| n (case/total) | 283/758 | 262/764 | 282/753 | 263/754 |
| Model 1[a] | Ref | 0.94 (0.72, 1.23) | 0.99 (0.76, 1.30) | 0.93 (0.71, 1.22) |
| Model 2[b] | Ref | 0.94 (0.72, 1.22) | 0.94 (0.72, 1.22) | 0.91 (0.69, 1.19) |
| Cu | ≤ 845.64 | 1845.65 ~ 945.24 | 945.25 ~ 1055.79 | ≥ 1055.80 |
| n (case/total) | 238/758 | 248/757 | 296/757 | 308/757 |
| Model 1[a] | Ref | 0.91 (0.69, 1.2) | 1.06 (0.81, 1.39) | 1.16 (0.87, 1.54) |
| Model 2[b] | Ref | 0.90 (0.69, 1.19) | 0.90 (0.69, 1.19) | 1.15 (0.87, 1.52) |
| Zn | ≤4778.14 | 4778.15 ~ 5578.83 | 5578.84 ~ 6481.80 | ≥ 6481.81 |
| n (case/total) | 263/758 | 251/757 | 277/757 | 299/757 |
| Model 1[a] | Ref | 0.86 (0.65, 1.13) | 1.05 (0.80, 1.38) | 1.13 (0.86, 1.48) |
| Model 2[b] | Ref | 0.84 (0.64, 1.10) | 0.84 (0.64, 1.10) | 1.11 (0.85, 1.45) |
| As | ≤ 2.65 | 2.66 ~ 4.45 | 4.46 ~ 7.06 | ≥ 7.07 |
| n (case/total) | 264/760 | 277/756 | 274/756 | 275/757 |
| Model 1[a] | Ref | 1.07 (0.82, 1.40) | 1.08 (0.82, 1.41) | 1.14 (0.85, 1.52) |
| Model 2[b] | Ref | 1.08 (0.83, 1.41) | 1.08 (0.83, 1.41) | 1.05 (0.80, 1.39) |
| Se | ≤ 138.60 | 138.61 ~ 165.50 | 165.51 ~ 195.45 | ≥ 195.46 |
| n (case/total) | 282/759 | 266/756 | 253/757 | 289/757 |
| Model 1[a] | Ref | 1.03 (0.79, 1.35) | 0.90 (0.69, 1.19) | 1.07 (0.81, 1.42) |
| Model 2[b] | Ref | 1.02 (0.79, 1.34) | 1.02 (0.79, 1.34) | 1.02 (0.78, 1.34) |
| Cd | ≤ 1.30 | 1.31 ~ 2.28 | 2.29 ~ 4.37 | ≥ 4.38 |
| n (case/total) | 240/760 | 252/755 | 303/758 | 295/756 |
| Model 1[a] | Ref | 1.13 (0.85, 1.50) | 1.23 (0.92, 1.64) | **1.49 (1.10, 2.01)** |
| Model 2[b] | Ref | 1.13 (0.85, 1.50) | 1.13 (0.85, 1.50) | **1.51 (1.12, 2.04)** |

| | | | | |
|---|---|---|---|---|
| Pb | ≤ 26.39 | 26.40 ~ 39.50 | 39.51 ~ 59.19 | ≥ 59.20 |
| n (case/total) | 191/758 | 269/757 | 303/757 | 327/757 |
| Model 1[a] | Ref | **1.34 (1.00, 1.79)** | **1.43 (1.07, 1.92)** | **1.55 (1.15, 2.09)** |
| Model 2[b] | Ref | **1.35 (1.01, 1.81)** | **1.35 (1.01, 1.81)** | **1.55 (1.15, 2.08)** |

[a] Model 1 was adopted the general logistics regression with the 11 districts as fixed effect and adjusted for age, sex, education level, drinking status, smoking status, family history of hypertension, anti-hypertensive use, BMI, TC, TG and FBG.

[b] Model 2 was adopted the mixed effects logistic regression, taking the 11 districts as a random intercept, and adjusted for age, sex, education level, drinking status, smoking status, family history of hypertension, anti-hypertensive use, BMI, TC, TG and FBG.

Bold indicates statistically significant ($P < 0.05$).

**Supplementary Table 3 Comparison of goodness of fit between fixed effect model and random effect model in 11 districts.**

| Whole blood metals | AIC | | *P* value |
|---|---|---|---|
| | Model 1[a] | Model 2[b] | |
| V | 2686.9 | 2688.0 | 0.025* |
| Cr | 2737.4 | 2740.3 | 0.013* |
| Mn | 2731.5 | 2735.0 | 0.011* |
| Co | 2735.6 | 2738.7 | 0.012* |
| Ni | 2739.2 | 2742.3 | 0.012* |
| Cu | 2736.6 | 2739.7 | 0.012* |
| Zn | 2735.3 | 2738.2 | 0.013* |
| As | 2738.8 | 2742.5 | 0.010* |
| Se | 2738.0 | 2741.3 | 0.011* |
| Cd | 2732.6 | 2735.0 | 0.016* |
| Pb | 2730.6 | 2733.7 | 0.012* |

[a] Model 1 was adopted the general logistics regression with the 11 districts as fixed effect and adjusted for age, sex, education level, drinking status, smoking status, family history of hypertension, anti-hypertensive use, BMI, TC, TG and FBG.

[b] Model 2 was adopted the mixed effects logistic regression, taking the 11 districts as a random intercept, and adjusted for age, sex, education level, drinking status, smoking status, family history of hypertension, anti-hypertensive use, BMI, TC, TG and FBG.

$^*P < 0.05$.

**Supplementary Table 4 Adjusted odds ratios (95%CI) for hypertension according to quartile of whole blood metals exposure.**

| Whole blood metals | Quartiles of whole blood metals (μg/L) | | | |
|---|---|---|---|---|
| | Q1 | Q2 | Q3 | Q4 |
| V | ≤ 0.52 | 0.53 ~ 0.98 | 0.99 ~ 1.55 | ≥ 1.56 |
| n (case/total) | 287/755 | 277/747 | 236/741 | 271/736 |
| Model 3[c] | Ref | 1.10 (0.82, 1.43) | 0.92 (0.70, 1.22) | 1.00 (0.76, 1.31) |
| Model 4[d] | Ref | 1.10 (0.85, 1.42) | 0.90 (0.68, 1.19) | 0.99 (0.75, 1.29) |
| Cr | ≤ 3.75 | 3.76 ~ 4.94 | 4.95 ~ 6.47 | ≥ 6.48 |
| n (case/total) | 286/760 | 272/758 | 258/756 | 274/755 |
| Model 3[c] | Ref | 1.05 (0.80, 1.36) | 0.92 (0.70, 1.21) | 0.97 (0.74, 1.26) |
| Model 4[d] | Ref | 1.03 (0.79, 1.34) | 0.91 (0.69, 1.19) | 0.96 (0.73, 1.25) |
| Mn | ≤ 10.79 | 10.80 ~ 13.96 | 13.97 ~ 17.99 | ≥ 18.00 |
| n (case/total) | 263/758 | 296/757 | 261/757 | 270/757 |
| Model 3[c] | Ref | **1.48 (1.14, 1.93)** | 1.26 (0.94, 1.61) | **1.42 (1.08, 1.86)** |
| Model 4[d] | Ref | **1.47 (1.13, 1.92)** | 1.23 (0.94, 1.61) | **1.41 (1.07, 1.85)** |
| Co | ≤ 0.14 | 0.15 ~ 0.28 | 0.29 ~ 0.39 | ≥ 0.40 |
| n (case/total) | 314/881 | 260/665 | 276/742 | 240/741 |
| Model 3[c] | Ref | 1.25 (0.97, 1.62) | 1.01 (0.78, 1.31) | 1.19 (0.91, 1.55) |
| Model 4[d] | Ref | 1.25 (0.97, 1.62) | 1.01 (0.78, 1.31) | 1.19 (0.91, 1.54) |
| Ni | ≤ 1.26 | 1.27 ~ 2.24 | 2.25 ~ 3.96 | ≥ 3.97 |
| n (case/total) | 283/758 | 262/764 | 282/753 | 263/754 |
| Model 3[c] | Ref | 0.97 (0.75, 1.26) | 1.02 (0.79, 1.33) | 0.89 (0.68, 1.15) |
| Model 4[d] | Ref | 0.97 (0.74, 1.25) | 1.02 (0.78, 1.32) | 0.89 (0.68, 1.15) |
| Cu | ≤ 845.64 | 1845.65 ~ 945.24 | 945.25 ~ 1055.79 | ≥ 1055.80 |
| n (case/total) | 238/758 | 248/757 | 296/757 | 308/757 |
| Model 3[c] | Ref | 0.93 (0.71, 1.22) | 1.0 (0.84, 1.43) | 1.13 (0.86, 1.48) |
| Model 4[d] | Ref | 0.93 (0.71, 1.22) | 1.09 (0.83, 1.43) | 1.13 (0.86, 1.48) |
| Zn | ≤4778.14 | 4778.15 ~ 5578.83 | 5578.84 ~ 6481.80 | ≥ 6481.81 |
| n (case/total) | 263/758 | 251/757 | 277/757 | 299/757 |
| Model 3[c] | Ref | 0.83 (0.63, 1.08) | 1.01 (0.77, 1.32) | 1.07 (0.82, 1.39) |
| Model 4[d] | Ref | 0.82 (0.63, 1.08) | 1.01 (0.77, 1.32) | 1.07 (0.82, 1.40) |
| As | ≤ 2.65 | 2.66 ~ 4.45 | 4.46 ~ 7.06 | ≥ 7.07 |
| n (case/total) | 264/760 | 277/756 | 274/756 | 275/757 |
| Model 3[c] | Ref | 1.12 (0.86, 1.45) | 1.18 (0.90, 1.55) | **1.41 (1.04, 1.91)** |
| Model 4[d] | Ref | 1.12 (0.86, 1.45) | 1.17 (0.90, 1.54) | **1.37 (1.05, 1.85)** |
| Se | ≤ 138.60 | 138.61 ~ 165.50 | 165.51 ~ 195.45 | ≥ 195.46 |
| n (case/total) | 282/759 | 266/756 | 253/757 | 289/757 |
| Model 3[c] | Ref | 0.99 (0.76, 1.0) | 0.86 (0.66, 1.13) | 1.06 (0.81, 1.39) |
| Model 4[d] | Ref | 1.00 (0.77, 1.30) | 0.86 (0.66, 1.13) | 1.05 (0.81, 1.38) |
| Cd | ≤ 1.30 | 1.31 ~ 2.28 | 2.29 ~ 4.37 | ≥ 4.38 |
| n (case/total) | 240/760 | 252/755 | 303/758 | 295/756 |
| Model 3[c] | Ref | 1.14 (0.86, 1.51) | 1.21 (0.91, 1.61) | **1.42 (1.05, 1.91)** |
| Model 4[d] | Ref | 1.14 (0.86, 1.51) | 1.21 (0.91, 1.61) | **1.46 (1.06, 1.93)** |

| | | | | |
|---|---|---|---|---|
| Pb | ≤ 26.39 | 26.40 ~ 39.50 | 39.51 ~ 59.19 | ≥ 59.20 |
| n (case/total) | 191/758 | 269/757 | 303/757 | 327/757 |
| Model 3[c] | Ref | 1.26 (0.94, 1.68) | 1.27 (0.95, 1.70) | **1.38 (1.02, 1.85)** |
| Model 4[d] | Ref | 1.27 (0.95, 1.70) | 1.29 (0.96, 1.73) | **1.40 (1.04, 1.88)** |

[c] Model 1 was adopted the general logistics regression with the region(coastal/inland) as fixed effect and adjusted for age, sex, education level, drinking status, smoking status, family history of hypertension, anti-hypertensive use, BMI, TC, TG and FBG.

[d] Model 2 was adopted the mixed effects logistic regression, taking the region(coastal/inland) as a random intercept, and adjusted for age, sex, education level, drinking status, smoking status, family history of hypertension, anti-hypertensive use, BMI, TC, TG and FBG.

Bold indicates statistically significant ($P < 0.05$).

**Supplementary Table 5 Comparison of goodness of fit between fixed effect model and random effect model in coastal/inland.**

| Whole blood metals | AIC | | *P* value |
|---|---|---|---|
| | **Model 3[c]** | **Model 4[d]** | |
| V | 2673.2 | 2679.8 | <0.001* |
| Cr | 2727.3 | 2734.1 | <0.001* |
| Mn | 2718.1 | 2725.2 | <0.001* |
| Co | 2724.0 | 2730.8 | <0.001* |
| Ni | 2726.9 | 2733.8 | <0.001* |
| Cu | 2725.9 | 2732.7 | <0.001* |
| Zn | 2724.1 | 2730.8 | <0.001* |
| As | 2723.1 | 2730.8 | <0.001* |
| Se | 2725.6 | 2732.6 | <0.001* |
| Cd | 2722.8 | 2729.3 | <0.001* |
| Pb | 2723.6 | 2730.0 | <0.001* |

[c] Model 1 was adopted the general logistics regression with the region(coastal/inland) as fixed effect and adjusted for age, sex, education level, drinking status, smoking status, family history of hypertension, anti-hypertensive use, BMI, TC, TG and FBG.

[d] Model 2 was adopted the mixed effects logistic regression, taking the region(coastal/inland) as a random intercept, and adjusted for age, sex, education level, drinking status, smoking status, family history of hypertension, anti-hypertensive use, BMI, TC, TG and FBG.

* $P < 0.05$.

**Supplementary Table 6 The correlations between whole blood metals among the study population.**

| | V | Cr | Mn | Co | Ni | Cu | Zn | As | Se | Mo | Cd | Tl | Pb |
|---|---|---|---|---|---|---|---|---|---|---|---|---|---|
| **V** | 1 | 0.318** | 0.001 | 0.011 | 0.009 | 0.047** | -0.075** | 0.213** | -0.180** | 0.032 | -0.054** | 0.219** | -0.024 |
| **Cr** | | 1 | 0.065** | 0.019 | 0.056** | -0.011 | 0.039* | 0.057** | 0.003 | 0.043* | -0.046* | 0.046* | -0.027 |
| **Mn** | | | 1 | 0.178** | 0.088** | 0.123** | 0.102** | 0.018 | 0.174** | 0.047** | 0.016 | -0.018 | 0.061** |
| **Co** | | | | 1 | 0.058** | 0.021 | -0.054** | 0.008 | 0.069** | -0.001 | 0.007 | -0.012 | -0.024 |
| **Ni** | | | | | 1 | 0.032 | 0.061** | -0.002 | 0.046* | 0.064** | -0.004 | -0.005 | 0.025 |
| **Cu** | | | | | | 1 | 0.166** | -0.02 | 0.160** | 0.061** | -0.063** | -0.014 | 0.011 |
| **Zn** | | | | | | | 1 | 0.103** | 0.219** | 0.063** | 0.146** | 0.080** | 0.087** |
| **As** | | | | | | | | 1 | -0.302** | 0.138** | 0.050** | 0.834** | -0.061** |
| **Se** | | | | | | | | | 1 | 0.037* | 0.013 | -0.412** | 0.152** |
| **Mo** | | | | | | | | | | 1 | 0.023 | 0.108** | 0.061** |
| **Cd** | | | | | | | | | | | 1 | 0.041* | 0.241** |
| **Tl** | | | | | | | | | | | | 1 | -0.111** |
| **Pb** | | | | | | | | | | | | | 1 |

*$P$ <0.05, ** $P$ <0.01.

**Supplementary Table 7 Adjusted odds ratio for incident hypertension risk in subgroups stratified by BMI.**

| Whole blood metals (μg/L) | Odds Ratios (95% CI) | | | | *P* trend[a] |
|---|---|---|---|---|---|
| | Q1 | Q2 | Q3 | Q4 | |
| Mn | ≤ 10.79 | 10.80 ~ 13.96 | 13.97 ~ 17.99 | ≥ 18.00 | |
| n (case/total) | 263/758 | 296/757 | 261/757 | 270/757 | |
| Low body weight | Ref | 2.37 (0.69, 8.10) | 0.68 (0.19, 2.41) | 1.49 (0.46, 4.87) | 0.305 |
| Normal | Ref | 0.61 (0.41, 0.92)* | 1.06 (0.73, 1.54) | 0.96 (0.65, 1.40) | 0.039* |
| Overweight | Ref | 0.84 (0.52, 1.36) | 1.09 (0.68, 1.73) | 0.78 (0.49, 1.24) | 0.839 |
| Obesity | Ref | 0.67 (0.27, 1.69) | 1.38 (0.56, 3.42) | 0.76 (0.30, 1.90) | 0.696 |
| As | ≤ 2.65 | 2.66 ~ 4.45 | 4.46 ~ 7.06 | ≥ 7.07 | |
| n (case/total) | 264/760 | 277/756 | 274/756 | 275/757 | |
| Low body weight | Ref | 0.64 (0.18, 2.23) | 0.61 (0.16, 2.26) | 1.04 (0.27, 3.94) | 0.943 |
| Normal | Ref | 1.22 (0.85, 1.77) | 1.04 (0.70, 1.54) | 1.01 (0.65, 1.59) | 0.950 |
| Overweight | Ref | 0.95 (0.59, 1.54) | 1.25 (0.78, 2.02) | 1.91 (1.13, 3.23)* | 0.014* |
| Obesity | Ref | 1.13 (0.42, 3.03) | 2.31 (0.87, 6.11) | 1.40 (0.47, 4.19) | 0.287 |
| Cd | ≤ 1.30 | 1.31 ~ 2.28 | 2.29 ~ 4.37 | ≥ 4.38 | |
| n (case/total) | 240/760 | 252/755 | 303/758 | 295/756 | |
| Low body weight | Ref | 1.84 (0.40, 8.40) | 1.61 (0.36, 7.22) | 2.30 (0.53, 9.98) | 0.312 |
| Normal | Ref | 1.36 (0.88, 2.10) | 1.50 (0.97, 2.31) | 1.85 (1.16, 2.94)* | 0.011* |
| Overweight | Ref | 0.79 (0.49, 1.25) | 0.80 (0.49, 1.32) | 0.98 (0.58, 1.66) | 0.971 |
| Obesity | Ref | 1.06 (0.43, 2.61) | 0.54 (0.20, 1.48) | 0.52 (0.17, 1.59) | 0.772 |
| Pb | ≤ 26.39 | 26.40 ~ 39.50 | 39.51 ~ 59.19 | ≥ 59.20 | |
| n (case/total) | 191/758 | 269/757 | 303/757 | 327/757 | |
| Low body weight | Ref | 1.06 (0.27, 4.13) | 1.10 (0.33, 3.67) | 1.85 (0.67, 5.10) | 0.980 |
| Normal | Ref | 0.93 (0.59, 1.47) | 1.14 (0.77, 1.69) | 1.16 (0.81, 1.67) | 0.902 |
| Overweight | Ref | 0.42 (0.25, 0.73)* | 0.72 (0.45, 1.15) | 0.60 (0.39, 0.95)* | 0.007* |
| Obesity | Ref | 1.40 (0.48, 4.09) | 1.12 (0.43, 2.93) | 0.98 (0.39, 2.47) | 0.517 |

Model was adjusted for age, sex, district, education level, drinking status, smoking status, family history of hypertension, anti-hypertensive use, TC, TG, FBG and four metals in the multi-metal model.

[a] *P* trend across quartiles of metals was obtained by including the median of each quartile (natural ln-transformed metals concentration) as a continuous variable in the model.

* $P < 0.05$.
